# Turbostratic Carbon/Graphene Prepared via the Dry Ice in Flames Method and Its Purification Using Different Routes: A Comparative Study

**DOI:** 10.3390/ma15072501

**Published:** 2022-03-29

**Authors:** Eduardo Cuadros-Lugo, Manuel Piñon-Espitia, Harby A. Martinez-Rodríguez, Daniel Lardizabal-Gutierrez, Ivanovich Estrada-Guel, Jose M. Herrera-Ramirez, Caleb Carreño-Gallardo

**Affiliations:** 1 Centro de Investigación en Materiales Avanzados—CIMAV, Miguel de Cervantes 120, Chihuahua 31136, Mexico; eduardo.cuadros@cimav.edu.mx (E.C.-L.); manuel.pinon@cimav.edu.mx (M.P.-E.); harby.martinez@cimav.edu.mx (H.A.M.-R.); daniel.lardizabal@cimav.edu.mx (D.L.-G.); ivanovich.estrada@cimav.edu.mx (I.E.-G.); 2 Grupo de Propiedades Térmicas Dieléctricas de Compósitos, Universidad Nacional de Colombia Sede Manizales, Manizales 170001, Colombia

**Keywords:** turbostratic carbon, graphene, dry ice, mechanical milling, magnesium oxide

## Abstract

Although the dry ice method used to synthesize turbostratic carbon/graphene is little known and used, it has significant advantages over others, such as the following: it is low cost, simple, and a large quantity of material can be obtained using some inorganic and highly available acids (which can be reused). Despite the above advantages, the main reason for its incipient development is the resulting presence of magnesium oxide in the final product. In the present work, three different treatments were tested to remove this remnant using some acid chemical leaching processes, including hydrochloric acid, aqua regia, and piranha solution. Based on the experimental evidence, it was determined that using aqua regia and combining the leaching process with mechanical milling was the most efficient way of removing such a remnant, the residue being only 0.9 wt.%. This value is low compared to that obtained with the other acid leaching solutions and purification processes (2.8–29.6 wt.%). A mandatory high-energy mechanical milling stage was necessary during this treatment to expose and dissolve the highly insoluble oxide without secondary chemical reactions on the turbostratic carbon. High-energy mechanical milling is an effective route to exfoliate graphite, which allows the magnesium oxide to be more susceptible to acid treatment. A yield of turbostratic carbon/graphene of 1 wt.% was obtained from the metallic Mg. The obtained surface area was 504.8 m^2^g^−1^; this high value resulting from the intense exfoliation can potentiate the use of this material for a wide variety of applications.

## 1. Introduction

Turbostratic graphite is composed of carbon layer structures that are not ordered as in the case of graphite, i.e., turbostratic carbon layers are not fully aligned parallel as in the case of graphite. It is well known that turbostratic carbon structures corresponding to graphene and exfoliated graphite can be identified if the stacking sequence is of few or many layers of carbon [1]. Although 16 years have passed since its discovery, graphene is still of great interest to the scientific community [2]. Its applications include sensors, composite strengthening [3], hydrogen storage, and lithium-ion batteries, among others. Since 2013, about 15,000 articles per year have been written on these topics. Today, several synthesis methods are well known, including the Hummer route [4], laser ablation, CVD [5], and high-energy milling [6]. Most of these methods involves serious disadvantages, such as environmental pollution concerns [7], due mainly to the use of toxic reagents like sulfuric acid [8], potent reducing agents like hydrazine [9], or hazardous aromatic solvents like toluene, benzene, etc. [10]. Some other methods need expensive equipment for their production or require the use of high-purity gases [11]. The synthesis method using carbon dioxide as a precursor, which is known as the dry ice in flames method, was developed by Chakrabarti and coworkers [12]. The method consists of igniting magnesium metal in a carbon dioxide (CO_2_) atmosphere using a block of dry ice. This results in a highly exothermic reaction, reaching a temperature above 1500 °C, enough to perform the following chemical reaction [13,14]:

2 Mg(s) + CO_2_ (g) → 2MgO (s) + C (s)

The obtained products are magnesium oxide (MgO) and carbon (C). The latter can be presented in different forms, from exfoliated graphite to graphene. While exfoliated graphite can have large clusters of hundreds of graphene layers, graphene is made up of few layers (~10). The main disadvantage of this method lies with the separation of magnesium oxide from exfoliated graphite and graphenes [15]. The predominant purification method reported in different studies consists of a chemical dissolution with hydrochloric acid (HCl), forming magnesium chloride (MgCl_2_), which is soluble in water, and its elimination by washing with demineralized water until a neutral pH is reached. The purified exfoliated graphite is dried [15,16]. Unfortunately, there is a problem related to a considerable amount of oxide (up to 5 at.%), which is impossible to remove, this remnant contamination being undesirable for some applications [17,18]. Although, this production method has numerous advantages, such as low production costs and the fact that the amount of obtained product is far greater than what can be obtained through other synthesis techniques [19]. Even when the process uses acid for purification, the washing waters are quickly neutralized, avoiding their corrosive nature. For all these reasons, some authors consider it an eco-friendly method [20,21].

The present study proposes different chemical purification routes of the dry ice in flames products because magnesium oxide is difficult to dissolve fully. For this purpose, three acid solutions (namely hydrochloric acid, aqua regia, and piranha solution) were tested, combining the leaching process with high-energy ball milling at reduced processing times. To follow the changes and processing differences, some samples were analyzed before and after the purification process through different characterization techniques, such as Raman spectroscopy, surface area analysis (BET), transmission electron microscopy (TEM), thermogravimetric analysis (TGA), and X-ray photoelectron spectroscopy (XPS).

## 2. Materials and Methods

### 2.1. Synthesis Method

**Figure 1:**
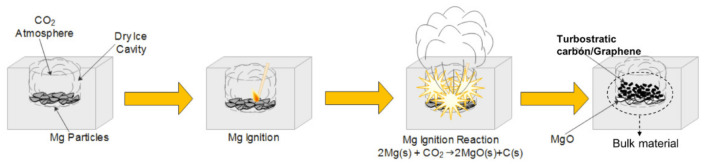
Descriptive diagram of the synthesis route to obtain turbostratic carbon/graphene.

The turbostratic carbon/graphene synthesis was carried out using a 20 cm solid cubic block of dry ice (solid carbon dioxide), to which a cavity 10 cm in depth and 7 cm in diameter was made. 20 g of pure magnesium chips (Sigma-Aldrich, San Louis, MO, USA, 6–35 mesh, 99.98% purity) were introduced into the cavity and the reaction was induced through a spark provided by a butane gas lighter (Figure 1). This operation was repeated until a total of 150 g was obtained; as mentioned, the obtained material consisted of a mixture of turbostratic carbon/graphene and magnesium oxide in powder form.

### 2.2. Purification Methods

The obtained powders were subjected to a chemical purification process based on three different acid solutions (Table 1).

**Figure 2:**
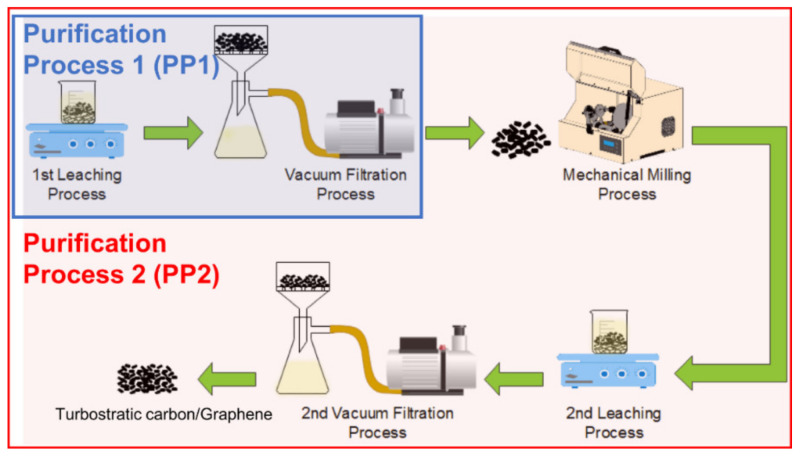
Illustrative diagram of sample purification processing.

A general scheme of the involved processes used to remove magnesium oxide is presented in Figure 2. Two purification processes were performed in the present study. The first process (PP1) consisted of leaching the bulk material synthesis product with the three solutions indicated in Table 1, followed by vacuum filtering. In the second process (PP2), the material obtained from PP1 was further processed by mechanical milling. It was then leached again with the same acid solution of the first leaching process, and finally it was vacuum filtered. The yield of turbostratic carbon/graphene was 1 wt.%, which is comparable to other traditional methods [22]. The obtained samples were characterized through the techniques mentioned above. The main reason to complement the leaching with high-energy ball milling is that mechanical milling causes the removal of the carbon layers that are firmly attached to the MgO. Consequently, it promotes an increase in chemical attacks, which enhance the dissolution of these unwanted particles. Also, the high-energy mechanical milling helps to promote the exfoliation of the graphite from unexfoliated graphite [23].

### 2.3. Leaching

The leaching process was set to 24 h for all samples under constant stirring at 80 °C [24].

### 2.4. Vacuum Filtering

After the leaching process, a vacuum filtration was carried out in the wet mixtures using a 500-mL Kitasato flask coupled with a Büchner funnel and a Whatman filter paper (number 42). The powders were washed with demineralized water until reaching neutral pH and dried on a laboratory stove at 100 °C overnight.

### 2.5. Milling

High-energy ball milling was carried out using a Spex 8000 M (Fisher Scientific, Metuchen, NJ, USA) device with a milling time of 30 min at 1427 RPM. 1 g of the washed and dried sample and six 13 mm steel-chromium coated balls were placed inside a 57 mL-capacity steel container. A ball-to-powder weight ratio was kept to 30:1 (in weight) for all experimental runs. Due to graphitic carbon’s lubricant and inert nature, no process control agent or argon atmosphere was used during the milling process.

### 2.6. Characterization Techniques

The presence of phases and their crystalline characteristics were studied by X-ray diffraction (XRD) in a Panalytical X’ Pert-Pro diffractometer (Anton Paar, Boynton Beach, FL, USA) working at 40 kV and 35 mA using Cu-Kα radiation with a wavelength of 0.154056 nm; the data were collected in the 2θ range from 5 to 80° at a scan rate of 0.2 deg/s. Thermogravimetric analysis (TGA) was performed using a TA Instrument model Q600 (TA Instrument, New Castle, DE, USA), with a heating ramp of 10 °C/min from room temperature to 800 °C under an airflow of 50 cm^3^/min. Raman spectrometry was performed using a LabRam HR VIS-633 microscope (HORIBA, Ltd. Miyanohigashi, Japan), equipped with a He-Ne laser source. Transmission electron microscopy (TEM) using a Hitachi 7700 microscope (HITACHI, Tokyo, Japan) and high-resolution transmission electron microscopy (HRTEM) using a TEM JEOL JEM 2200FS (JEOL, Tokyo, Japan) microscope was employed to analyze the synthesized turbostratic carbon/graphene. For the TEM sample preparation, 0.10 mg of each sample was weighed and placed in a glass vial with 4 mL of isopropyl alcohol and sealed tightly. It was subjected to an ultrasonic agitation (Branson Digital Sonifier, Danbury, CT, USA) for 15 min. Later, a drop of the sonicated solution was taken with a capillary tube and deposited on a copper grid with a “Lacey Formvar/carbon” membrane. Subsequently, it was placed under an IR lamp for 15 min to dry the sample. Finally, the sample was taken to a plasma cleaner system to remove any impurities. The identification of the Miller indices was made by the selected area electron diffraction (SAED) patterns. N_2_ adsorption–desorption isotherms were obtained by the Brunauer–Emmett–Teller (BET) method in a Quanta chrome model Nova 4200e analyzer (Anton Paar, Boynton Beach, FL, USA), taking 11 points of 0.05 to 0.3 of relative pressure (P/P_0_); from this analysis, the surface area and the pore distribution were determined. The XPS study was carried out on a Thermofisher XPS Escalab 250Xi (Thermo Scientific, Waltham, MA, USA) device under the following conditions: 10 eV scanning energy, 0.1 eV resolution, dwell time of 200 ms, 40 scanning times per spectrum, and an angle of 90°. A monochromatic Al source (Kα1 = 1486.7 eV) was used in the analysis chamber; the materials were deposited on a graphite tape, adhered to the sample holder of the equipment, introduced into the pre-chamber at a vacuum pressure of 10^−6^ Torr, and finally taken to the analysis chamber at a pressure of 10-10 Torr. The monochromator was located at an angle of 45° (2χ) with respect to the source. The C 1s and Mg 1s spectra were deconvoluted using the Aanalyzer^®^ software, (RDATA, v1.45, Queretaro, México) and the peak fitting was performed using Doniach-Sunjic-Shirley (DSS) functions.

## 3. Results

### Characterization of Turbostratic Carbon/Graphene Powders

**Figure 3:**
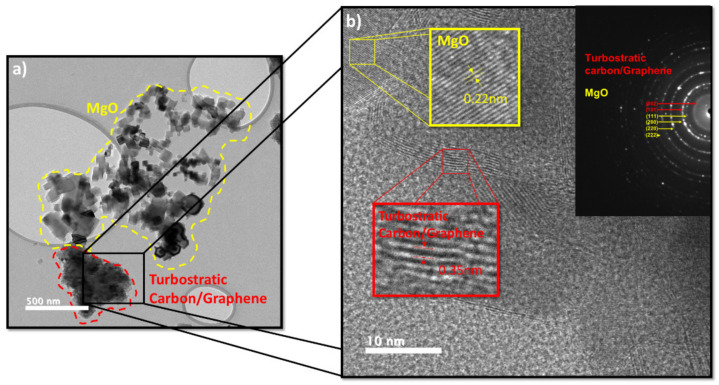
(**a**) TEM image (**b**) HRTEM image and SAED pattern of the raw sample obtained after the synthesis.

TEM studies were carried out to determine the phase coexistence, particle size, morphology, and microstructure of samples. The obtained material from the dry ice synthesis process without any additional processing was analyzed. Figure 3a shows a TEM micrograph where a mixture of turbostratic carbon structures is observed, and the presence of a considerable concentration of MgO in the form of cubical particles is also demonstrated. Figure 3b shows a HRTEM micrograph where a notable difference in both materials can be observed by the measurement of the interplanar distance: 0.35 nm [25,26] for turbostratic carbon/graphene and 0.22 nm for MgO. The SAED pattern in Figure 3b indicates the presence of both MgO and turbostratic carbon/graphene. The proposed purification processing methods aim to remove these agglomerated and exposed particles.

**Figure 4:**
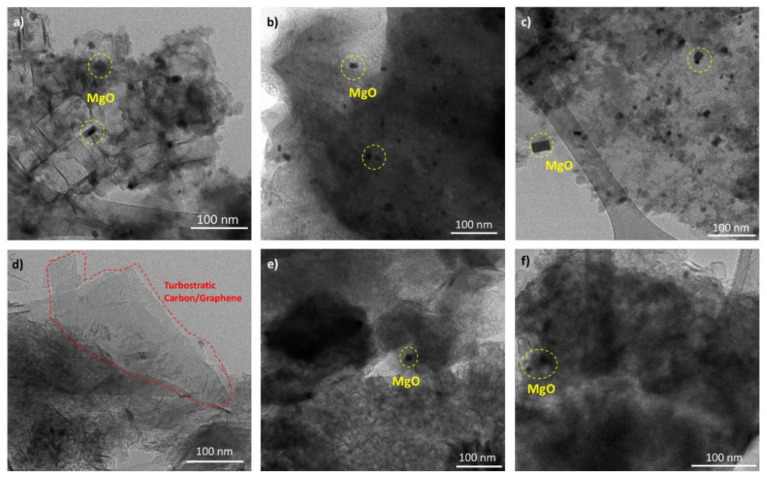
HRTEM images of (**a**) LM1-PP1, (**b**) LM2-PP1, (**c**) LM3-PP1, (**d**) LM1-PP2, (**e**) LM2-PP2, and (**f**) LM3-PP2 samples.

Figure 4 shows HRTEM micrographs of samples after the purification processes. Micrographs in Figure 4a–c correspond to samples processed by the first purification process (PP1), where the presence of sheets and embedded MgO nanoparticles is clear. These nanoparticles have characteristically cubic geometry, for which size dispersion varies from 10 to 20 nm. Micrographs in Figure 4d–f correspond to samples subjected to the first purification process (PP1) followed by mechanical milling and a second purification process (PP2) to remove the MgO phase as much as possible. These images show clear evidence of turbostratic carbon sheet (Figure 4d), where the van der Waals interlayer attractions allowed the nanosheets to slide over each other perpendicularly to the c-axis. Still, enough attraction prevents the complete formation of individual graphene monolayers; the diffraction contrast is related to the thickness variation, denoting the presence of multiple turbostratic carbon layers and polycrystalline structures with randomly oriented grains. After the second leaching process, the relative concentration of MgO particles decreased considerably compared to samples from the first leaching process.

**Figure 5:**
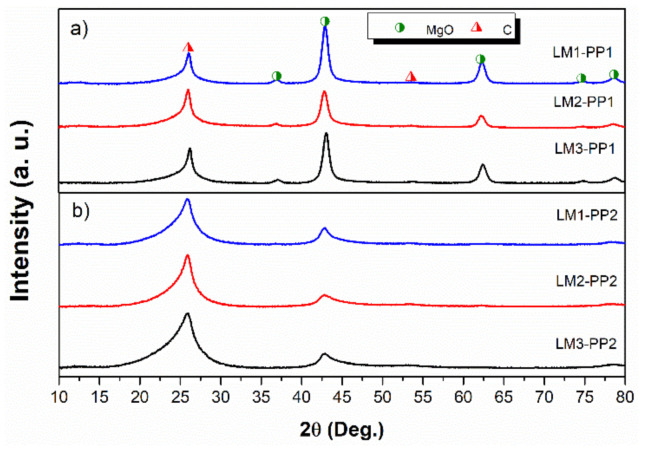
XRD patterns of (**a**) PP1 and (**b**) PP2 samples.

Comparative X-ray diffraction patterns of samples under different processing conditions are displayed in Figure 5. Figure 5a presents the diffractograms of samples after the first purification process. The indexed diffraction peaks located at 2θ ≈ 26 and 54° are attributed to the planes (0 0 2) and (0 0 4), respectively, both corresponding to a turbostratic structure (JCPDS 41-1487). The signals at 37, 43, 63, 75, and 78° are correlated with the planes (1 1 1), (2 0 0), (2 2 0), (3 1 1), and (2 2 2), respectively, and are attributed to MgO (periclase) (JCPDS 78-0430). Figure 5b presents the diffractograms of samples after the second purification process; although the MgO is still present, a significant decrease in its signals is evidenced. Due to the instrumental restriction of XRD, this analysis does not allow us to differentiate which purification method is better quantitatively.

The number of layers along the c-axis (N_c_) of graphene samples was calculated by the equation described by Seehra et al. [27] (Equation (1)), where L_c_ is the apparent crystallite size, which was determined from the Scherrer equation (Equation (2)) and d_002_ is the interplanar spacing of for the (002) plane, which was calculated from the Bragg’s Law (Equation (3)) [28,29].

(1) $$Nc=\frac{L_{c}}{d_{002}}$$

(2) $$L_{c}=\frac{k\lambda }{\beta \cos\theta }$$

(3) $$d_{002}=\frac{n\lambda }{2\sin\theta }$$

As shown in Table 2, the number of layers decreases after the purification process 2.

**Figure 6:**
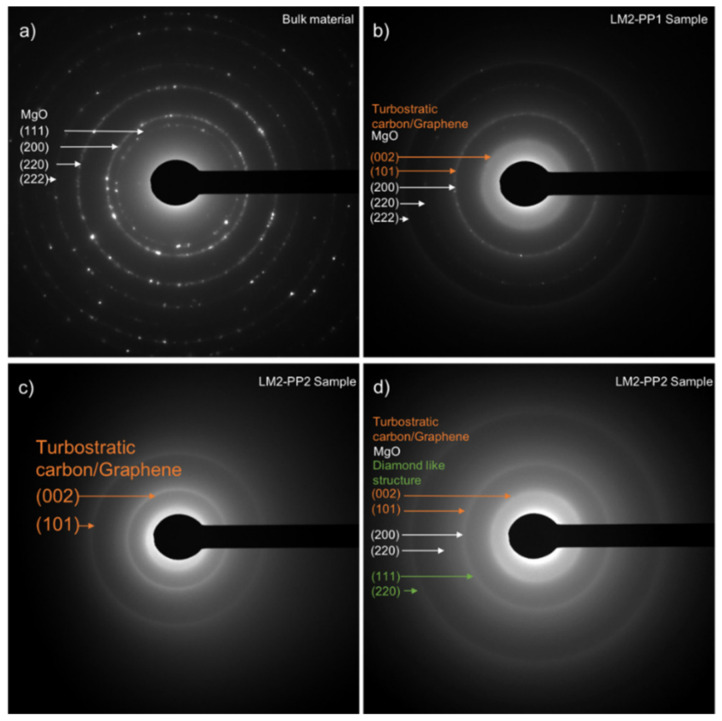
SAED patterns of (**a**) bulk material, (**b**) LM2-PP1 sample, (**c**) and (**d**) LM2-PP2 sample analyzed in different areas.

Figure 6 shows the SAED pattern analysis of the samples during their different processing stages. Figure 6a corresponds to the material obtained from magnesium and carbon dioxide reaction (bulk material ignition), in which the rings correspond to the MgO planes (1 1 1, 2 0 0, 2 2 0, and 2 2 2) are observed. Figure 6b corresponds to the LM2-PP1 sample, showing the main turbostratic carbon/graphene (0 0 2, 1 0 1) and MgO planes. An overlap between the (1 0 1) turbostratic carbon/graphene and (2 0 0) MgO planes is observed. The presence of MgO correlates with the coexistence of the two materials reported in XRD analysis. Figure 6c–d correspond to the SAED patterns in different analysis zones of the LM2-PP2 sample. In Figure 6c, the rings corresponding to the graphite planes are observed, but not magnesium oxide. Figure 6d shows both the graphite and magnesium oxide planes and (1 1 1) and (2 2 0) planes, corresponding to a diamond structure regarding the diffraction pattern with number JCPDS 6-0675, which was originated by the mechanical milling process and second washing.

**Figure 7:**
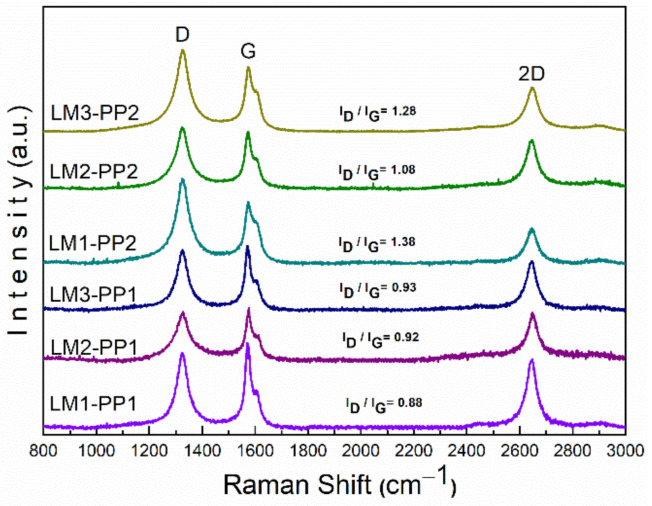
Comparative Raman spectra of PP1 and PP2 leached samples.

Figure 7 displays the results of Raman spectroscopy. The Raman spectra demonstrate that all samples are composed of a carbonaceous matrix with representative signals related to graphitic structures; these signals are commonly described as D, G, and 2D bands, which were detected at 1350, 1580, and 2600 cm^−1^, respectively [30]. As it is well known, the D band is related to lattice disorder and sp3 defects in graphenes, while the G band is the result of in-plane C-C symmetric stretching vibrations and is associated with the sp2 structure of carbon [31]. The 2D band is correlated with the overtone of the D band [32]. The D band and G band (I_D_/I_G_) intensity ratio is used to evaluate the degree of disorder and defects in the graphitic structure. As can be observed, the I_D_/I_G_ ratio was affected by the mechanical milling process increasing from 0.91 (average of the three leaching processes) for the first purification process (PP1) to 1.24 for the second purification process (PP2), which involves mechanical milling, leading to a yield of 99% from the PP2. The intensity of the D band at 1322 cm^−1^ of samples purified through the PP2 process is greater than that of PP1 samples. This intensity increment evidences that defects increased due to the mechanical milling process. Consequently, an increase in destructive exfoliation was produced and correlated with the broadening and decreasing intensity observed in the X-ray patterns (Figure 5).

The intensity of the 2D bands is lower for the PP2 samples than PP1, which can be attributed to defects in the graphene structure [33,34,35]. This decrease in intensity is characteristic of the disorder in the c-axis [36,37] and turbostratic structure formation derived from the disordered graphene layer arrangement resulting from the chemical reaction [13]. Also, the 2D band is inextricably linked to the electronic band structure of graphene and is a good indicator of a more graphene-like structure; this can be confirmed with the XPS results by the presence of the sp2 bonds, as will be demonstrated below [38].

**Figure 8:**
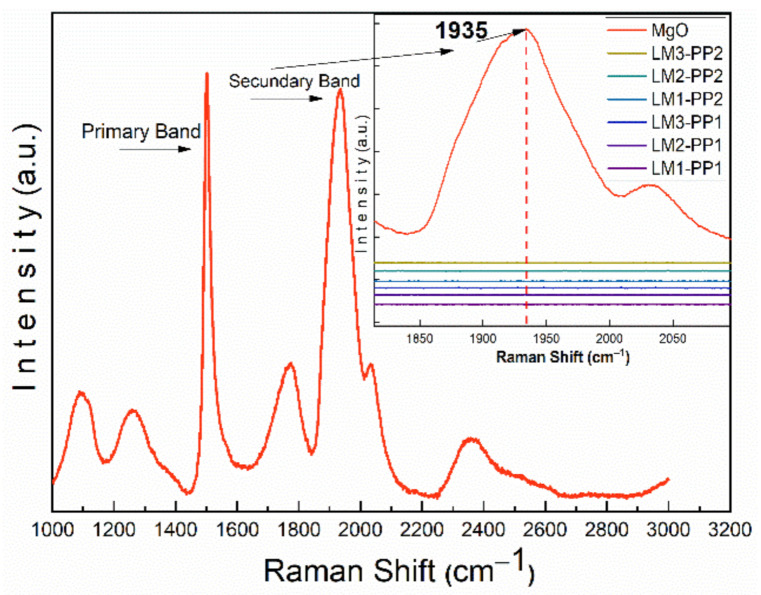
Comparative Raman spectra of MgO and leached samples.

A Raman analysis of MgO was performed to discard the contribution of its bands. The MgO spectrum is dominated by two main bands of approximately the same intensity, located at 1500 and 1935 cm^−1^ [39]. As illustrated in Figure 8, the 1500 cm^−1^ MgO band could overlap with the G band of graphene. However, when comparing the MgO spectrum with those of processed samples (inset in Figure 8), the presence of the 1935 cm^−1^ MgO band is not observed. Based on these findings, it can be assumed that there is no contribution of MgO bands in the analyzed samples. Therefore, it can be expected that the result of the I_D_/I_G_ ratio calculation is not affected. The split shape in the G band can be seen in all the samples and is attributed to the D*’* band located at ~1610 cm^−1^. Defects cause the appearance of the D*’* band in the carbon structure in all the analyzed samples [40,41].

**Figure 9:**
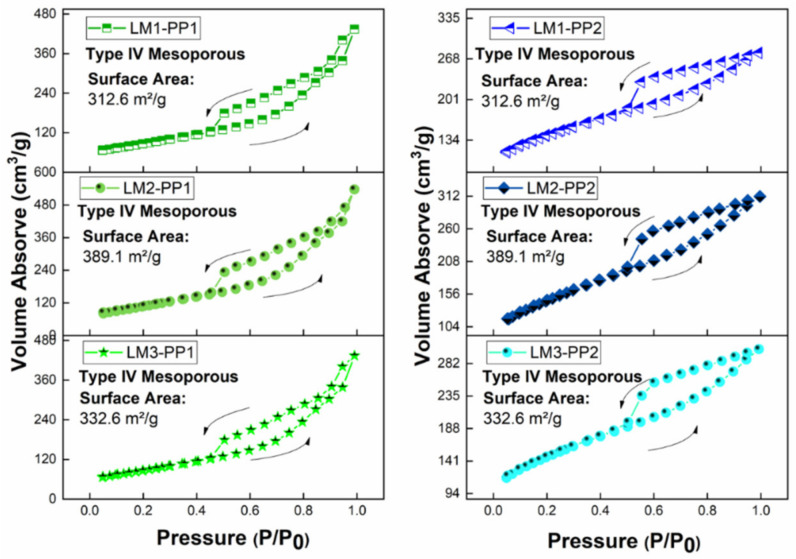
BET N_2_ adsorption-desorption isotherms and hysteresis loop: (**left**) PP1 and (**right**) PP2 samples.

Figure 9 presents the results of the BET analysis. The isotherms of both the PP1 and PP2 samples demonstrate a Type IV (mesoporous) structure, according to the BDDT classification. The amount of adsorption is less in the low-pressure area, increasing sharply in the high-pressure area. Likewise, according to the Boer definition, the PP1 and PP2 solid solutions present a Type B hysteresis loop, showing the same behavior in all the samples. This phenomenon is related to graphene materials, where the pores correspond to the spaces between the graphite sheets. A wide pore distribution was calculated (60–180 Å in diameter), which is related to a heterogeneous pore diameter of the materials [42]. One of the main effects of the solid adsorbent materials is presented in the interface, which generates the adsorption [43].

Table 3 presents the surface area (SA) analysis attained through the BET method. The values for PP1 samples are in the range of 300–390 m^2^g^−1^, while the PP2 samples presented higher and uniform values in the range of 500–505 m^2^g^−1^. With this, it can be pointed out that SA is a dependent variable closely related to the purification treatment.

**Figure 10:**
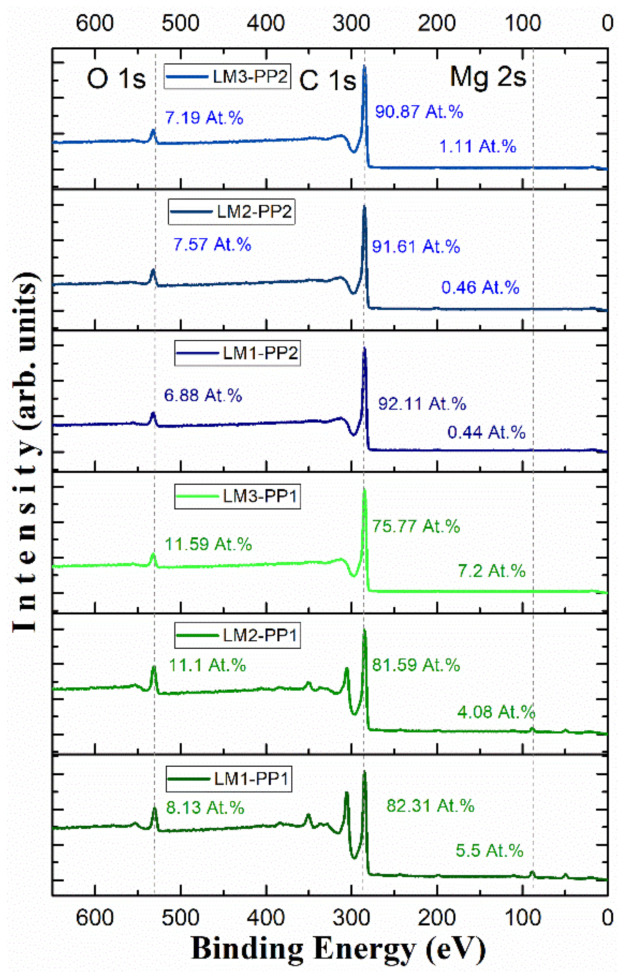
XPS survey spectra of LM1, LM2, and LM3 of PP1 and PP2 samples.

**Figure 11:**
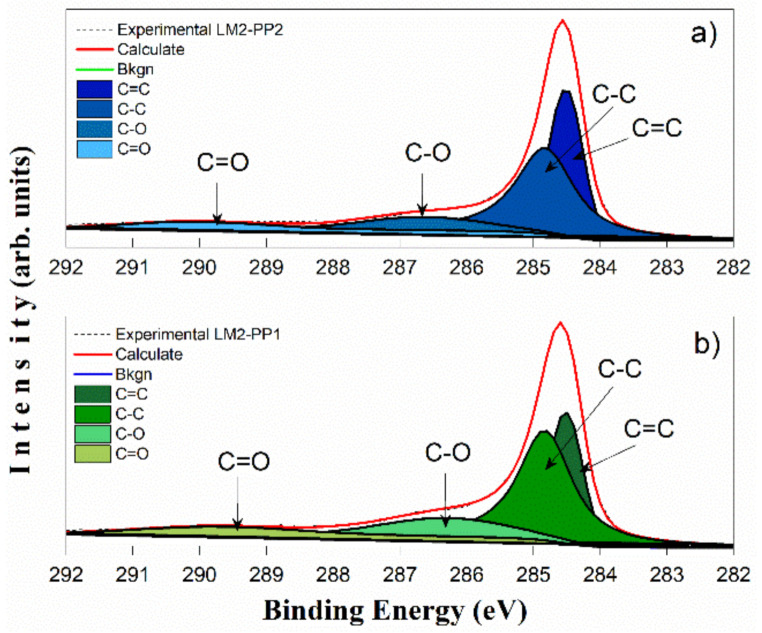
C 1s X-ray photoelectron spectra of the PP1 (**a**) and PP2 (**b**) processing of LM2 samples.

X-ray photoelectron spectroscopy was performed to quantify present elements that characterize the graphene sheets and the oxygenated functional groups retained in the samples after the leaching process. Figure 10 shows the X-ray photoelectron spectra of LM1, LM2, and LM3 samples after the PP1 and PP2 processes. Only the C 1s, O 1s, and Mg 2s core level signals are detected at binding energies of hv = 285 eV, 532 eV, and 88.47 eV, respectively. The PP1 samples show higher oxygen contents (10.2 at.% on average) than the PP2 samples with 7.1 at.%. This difference is related to the chemical reduction of MgO. XPS analysis shows a drastic magnesium decrease after the second process, leaving 0.67 at.%. This remnant is probably due to Mg trapped within the unleached carbon matrix. The survey shows the highest signal intensity for carbon C 1s; this peak is asymmetric due to the conjunction of the C=C and C-C signals, characteristic of turbostratified materials, corresponding to the sp2-hybridized carbon atoms and the distorted lattice carbon atoms with a single bond (sp3), respectively [44,45]. Four peaks corresponding to carbon atoms were identified. Two signals corresponding to nonfunctionalized areas of the turbostratic carbon lattice (peaks C=C at hv = 284.5 eV and C-C at 285.0 eV), and the other two are related to carbon atoms’ bond with oxygen (peaks C-O at 286.2 eV and C=O at 289.5 eV); these signals are better defined after deconvolution of the C 1s spectra. Figure 11 shows the XPS spectra for samples leached with aqua regia with both purification processes: LM2-PP2 (Figure 11a) and LM2-PP1 (Figure 11b). It can be observed that the PP2 acid leaching process does not increase the signals corresponding to oxidized species drastically. This allows us to assume the presence of turbostratic carbon [24]. Conversely, for the sample leached with hydrochloric acid with the first purification process (LM2-PP1), the peak of the C=O band was found at 289.5 eV. The C-O signal increased by 1.1% for the oxidized species, while the C=O signal decreased by 1.1%. We believe that this change was due to the chemical attack induced by the second leaching. The presence of oxidized groups is related to the different acid treatments; it is worth mentioning that the dry ice in flames method produces graphenes that do not start from oxidized graphite (GO) reduction processes as other synthesis methods do. However, a small concentration of oxidized groups is present from the synthesis and leaching acid treatments. The area percentage corresponding to each type of bond was determined, and the results are summarized in Table 4. It is observed that except for sample LM1, which did show a reduction of the oxidized groups, the other samples retained the same ratio of unoxidized to oxidized fractions. This reaffirms that the second leaching process does not cause oxidation in the samples.

Conversely, this table shows that carbon is mainly found with sp3 hybridization. This is corroborated by Raman analysis; meanwhile, the sp2 graphitic part contributes about 25% of the sample. A chemical reduction is observed in all the samples of the oxidized groups after the second treatment (PP2), which presents an average of ~17.3%, compared with ~22.9% for samples after the first treatment (PP1).

**Figure 12:**
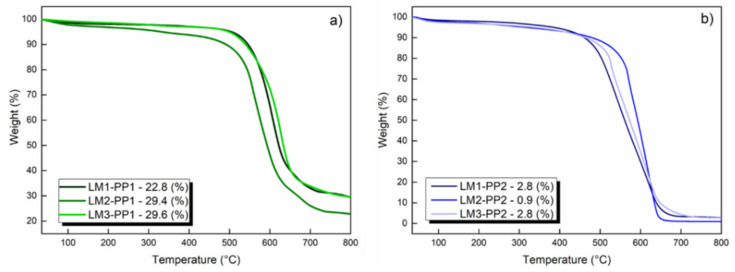
Comparative thermograms and residues of leached samples PP1 (**a**) and PP2 (**b**).

The thermogravimetric analysis (TGA) curves show the mass loss of samples as a function of the temperature (Figure 12). The mass loss related to carbon oxidation occurs between 500 and 750 °C, leaving a solid residue mainly composed of magnesium oxide. The final residue percentage of PP1 samples is between 22.8 and 29.6%, which is correlated as MgO (Figure 12a). This large amount of residue is why this synthesis method has not been widely developed. For PP2 samples (Figure 12b), the final residue percentage ranges from 0.9 to 2.9%, highlighting the leaching process where aqua regia is used (LM2) with a residue of only 0.9%. Aqua regia is a very effective solvent for inorganic materials because its two components (nitric acid and hydrochloric acid) act in a complementary way due to the combined effect of H^+^, NO^3−^, and Cl^−^ ions in solution. TGA analysis is a fundamental tool to quantitatively evaluate the best purification treatment (related to residue generation after calcination) [46].

## 4. Discussion

**Figure 13:**
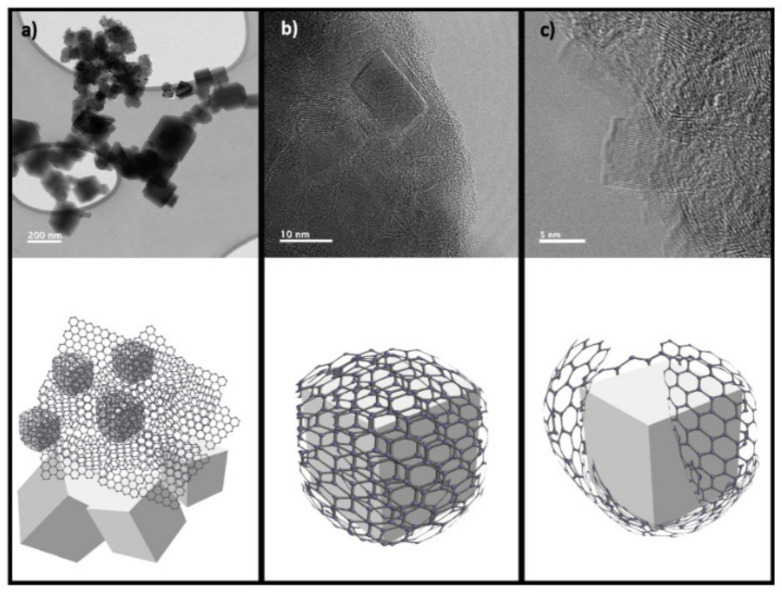
TEM micrographs and proposed dissolution mechanism. MgO nanoparticles surrounded by graphite (**a**), (**b**) MgO nanoparticles covered with several graphene layers and (**c**) graphite exfoliation by mechanical milling.

Based on the evidence obtained by TEM, a mechanism for the high concentration of unreacted MgO during the dry ice in flames synthesis method is proposed, which is schematized in Figure 13. Figure 13a shows a general view of the MgO nanoparticles surrounded by graphite. The TEM image in Figure 13b evidences that the MgO nanoparticles are covered with several graphene layers, preventing physical contact with the leaching chemical which would achieve their complete dissolution. These layers may be present in different thicknesses spanning a few layers; it can induce graphite formation if the material reaches many layers. The mechanical milling process promotes graphite exfoliation and induces an increase of surface exposure of the oxide; this facilitates the acid attack and the subsequent dissolution process (Figure 13c).

**Figure 14:**
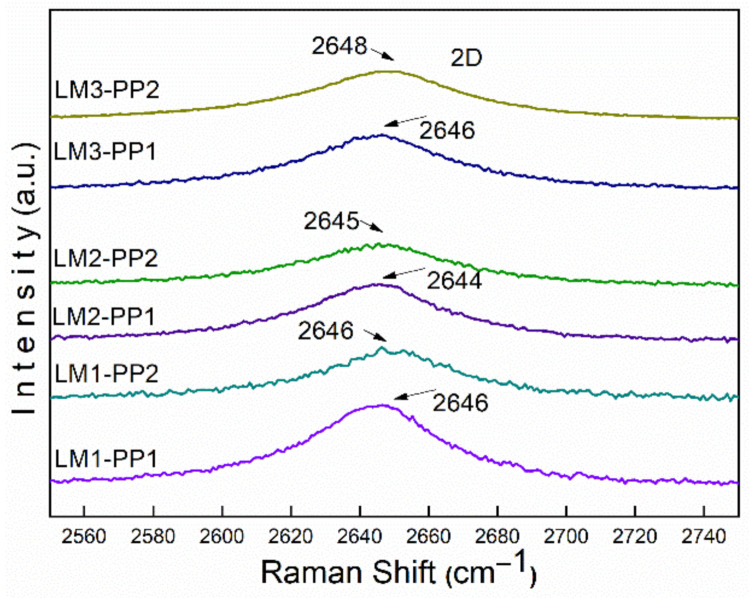
2D band comparison of the processes PP1 and PP2.

The sp2-type carbon materials exhibit a Raman spectrum with a strong peak in the 2500 to 2800 cm^−1^ range, corresponding to the 2D band. This is a second-order two-phonon process, which strongly depends on the used frequency of the laser energy excitation [47]. The 2D band can be used to determine the number of graphene layers. This is mainly applied to multilayer graphene structures, where the shape of the 2D band is quite different from that of single-layer graphene with more intense and sharper bands. In our case (Figure 14), the 2D band does not present significant differences, neither in the peak shape nor in the shift value between the PP1 and PP2 wash process, which was less than 3 cm^−1^. In addition, no shoulders, overlaps, or 2D1 and 2D2 bands can be observed. However, the I_G_/I_2D_ ratio has a contradictory behavior to the theoretical one. This behavior could be related to the synthesized material since it does not come from natural crystalline graphite, as it does in most studies. It is necessary to carry out future work to verify this behavior. We can say that the material is exfoliated as the high surface area results demonstrated, as reported in Table 3.

It is worth mentioning that van der Waals forces play an important role, as they are responsible for linking these graphene sheets together during the graphite formation. Based on the surface area results, it can be assumed that most of the particles remain as turbostratic carbon/graphene consisting of a few hundred graphene layers. Note that the theoretical surface area value for graphene is 2700 m^2^g^−1^ [48], which is well above the values found for the materials synthesized in this investigation; this may indicate how far we are from generating a graphene monolayer.

Another clear advantage of this purification process is the processing time to obtain a relatively elevated surface area (~500 m^2^g^−1^). Indeed, such a value of the surface area is achieved after 16 h when using high-energy ball milling obtained from natural graphite [23,49,50]. In contrast, the time used to carry out the exfoliation in this work was only 30 min, which is reflected in a considerable saving of time and can produce a greater quantity of material. Furthermore, our SA value is almost doubled compared to synthesis methods like reducing graphene oxide, where a surface area of 298.2 m^2^g^−1^ was obtained [51].

## 5. Conclusions

Turbostratic carbon/graphene was successfully prepared via the dry ice in flames method; a yield of 1 wt.% was achieved. This method can be a potential alternative to perform this purification process due to its low cost, simplicity, high surface area (500–505 m^2^g^−1^), and high scalability. The proposed mechanical milling stage and a second leaching process (PP2) are essential for the effective removal of MgO. A broad 2D Raman band showed structures conformed with a few layers of turbostratic carbon/graphene during the characterization. Under experimental conditions, no components other than carbon and oxygen were found in the samples. Before the PP2 process, the LM1, LM2, and LM3 samples showed visible XRD peaks corresponding to MgO; however, a remarkable decrease of the peaks was observed in the samples after the second purification process (PP2). Additional studies corroborated the presence of MgO; this phase was identified during TEM and SAED characterization. According to the experimental results obtained by the thermogravimetric analysis (TGA), the treatment using aqua regia in the PP2 process (LM2-PP2) was the most effective, reaching a value of 0.9 wt.% of MgO residues; these values are concordant with those obtained by XPS. Studied samples show mostly Sp3 and Sp2 bonds corresponding to the turbostratic structures. Based on experimental evidence, acid leaching processes do not contribute to carbon oxidation. The present route (aqua regia) is more effective than hydrochloric acid (LM1) treatment, which is universally used for this subject. The study opens the doors for using the purified exfoliated graphite/graphene for various applications where a high surface area is necessary, such as in catalysis and removal of solvents and heavy metals. The surface area analysis concluded that mechanical milling helps increase the surface area in the analyzed samples, thus obtaining a turbostratic carbon/graphene of greater purity.

## Figures and Tables

**Table 1:** Acid leaching solutions used during the purification processing.

Name	Composition	Nomenclature
Hydrochloric acid	HCl 1M	LM1
Aqua regia	HNO_3_:HCl 3:1 (in vol.)	LM2
Piranha solution	H_2_SO_4_:H_2_O_2_ (30 vol.%) 3:1	LM3

**Table 2:** Numbers of layers in turbostratic carbon samples.

Sample	Number of Layers
LM1-PP1	32
LM2-PP1	26
LM3-PP1	31
LM1-PP2	23
LM2-PP2	13
LM3-PP2	26

**Table 3:** Summary chart of SA of samples after leaching steps.

Sample	Surface Area (m^2^g^−1^)
LM1-PP1	332.6 ± 1
LM2-PP1	389.0 ± 1
LM3-PP1	312.6 ± 1
LM1-PP2	504.8 ± 1
LM2-PP2	503.4 ± 1
LM3-PP2	502.0 ± 1

**Table 4:** Summary chart with area percentages of the C 1s XPS spectra for the different treatments.

Binding	LM1-PP1	LM2-PP1	LM3-PP1	LM1-PP2	LM2-PP2	LM3-PP2
C=C	24.52	25.01	24.62	26.75	26.68	27.72
C-C	40.18	51.9	53.43	52.2	50.3	50.25
C-O	26.75	13.89	13.39	13.15	14.97	13.92
C=O	8.55	9.2	8.56	7.9	8.05	8.11

## Data Availability

The data presented in this study are available on request from the corresponding author. The data are not publicly available due to an ongoing research.
